# In Situ Fabrication of Bacterial Cellulose–Polyhydroxyalkanoate Composite Membranes from Coconut Water: Mechanical Performance and Life Cycle Assessment

**DOI:** 10.3390/gels12080675

**Published:** 2026-07-29

**Authors:** Panuvit Rienpradub, Suphuttra Boonmak, Anuchan Panaksri, Sani Boonyagul, Patnarin Worajittiphon, Woradej Pichaiaukrit, Ghit Laungsopapun, Prihartini Widiyanti, Korawan Sringarm, Pornchai Rachtanapun, Kittisak Jantanasakulwong, Nuttapol Tanadchangsaeng

**Affiliations:** 1 College of Biomedical Engineering, Rangsit University, Lak Hok, Muang, Pathum Thani 12000, Thailand; fern.panuvit@gmail.com (P.R.); b.suphuttra@gmail.com (S.B.); anuchan.p@rsu.ac.th (A.P.); sani@rsu.ac.th (S.B.); 2 Department of Chemistry, Faculty of Science, Chiang Mai University, Suthep, Mueang, Chiang Mai 50200, Thailand; patnarin.w@cmu.ac.th; 3 College of Dental Medicine, Rangsit University, Lak Hok, Muang, Pathum Thani 12000, Thailand; woradej.p@rsu.ac.th; 4 Material Properties Analysis and Development Center, Thailand Institute of Scientific and Technological Research, Khlong Ha, Khlong Luang, Pathum Thani 12120, Thailand; ghit@tistr.or.th; 5 Master of Biomedical Engineering Study Program, Faculty of Science and Technology, Universitas Airlangga, Surabaya 60115, Indonesia; pwidiyanti@fst.unair.ac.id; 6 Department of Animal and Aquatic Sciences, Faculty of Agriculture, Chiang Mai University, Suthep, Mueang, Chiang Mai 50200, Thailand; korawan.s@cmu.ac.th; 7 School of Agro-Industry, Faculty of Agro-Industry, Chiang Mai University, Mae Hia, Muang, Chiang Mai 50100, Thailand; pornchai.r@cmu.ac.th (P.R.); jantanasakulwong.k@gmail.com (K.J.)

**Keywords:** bacterial cellulose, polyhydroxyalkanoate, composite gels, response surface methodology, physical properties, tissue regeneration membrane, life cycle assessment

## Abstract

Bacterial cellulose (BC) is a highly pure biomaterial that can be produced from agro-industrial residues, making it a sustainable candidate for tissue engineering applications such as biocompatible hydrogel. However, pure BC is rigid and brittle, which limits its clinical handling. This study aimed to synthesise BC membranes from the non-photosynthetic bacterium *Komagataeibacter nataicola* (TISTR 975) using mature coconut water as the primary fermentation medium, and to improve its mechanical properties by forming an in situ composite hydrogel with polyhydroxyalkanoate (PHA). Sucrose concentration and cultivation period were systematically varied and analysed using response surface methodology (RSM), which identified the optimal condition as a 7-day cultivation at 50 g/L sucrose, yielding a highly uniform membrane with the best structural integrity. In situ fabrication of the BC/PHA composite, achieved by dispersing PHA powder in the culture medium during synthesis, markedly enhanced the material’s flexibility and water-holding capacity. The elongation at break increased from 22.9% for pure BC to 28.2% for the composite, with only a slight reduction in ultimate tensile strength. A cradle-to-gate life cycle assessment (LCA) showed that the BC/PHA membrane had a global warming potential approximately 3.2 times lower than that of bovine collagen membranes, while the incorporation of PHA introduced a negligible additional environmental burden. These findings indicate that the locally producible and biodegradable BC/PHA composite hydrogel offers a favourable balance between mechanical performance, water-holding capacity, and environmental sustainability, positioning it as a promising candidate for the future development of guided tissue regeneration (GTR) gel membranes in dentistry.

## 1. Introduction

Cellulose is a structural polysaccharide consisting of linear chains of beta-linked D-glucose units [1,2], and is essential for giving rigidity and mechanical strength to the plant cell wall matrix [3]. In addition to its natural function in structural support, plant cellulose is increasingly being used for the creation of biomedical materials such as wound dressings, drug delivery matrices, and bio-sustainable tissue engineering scaffolds based on a bio-sustainable material [4,5]. Plant-derived cellulose is usually extracted and purified from plant material using extensive chemical processes that remove lignin, hemicellulose, and other impurities that are not compatible with bioconversion or difficult to process, unlike the naturally occurring cellulose that bacteria can ferment [6,7,8]. These cellulose derivatives are then often functionalized to produce surface properties that are desirable for a variety of surface applications, such as structural integrity and high mechanical stability, for which they are suitable for clinical use. However, even after purification, these materials suffer from intrinsic poor crystallinity, larger and more heterogeneous fiber dimensions, and limited porosity that can hinder the infiltration of required cells and transport of nutrients to and within the scaffold [4,9].

Recently, the focus has shifted to bacterial cellulose (BC) as an alternative to cellulose produced by plants, as it bypasses the limitations of plant cellulose production [10,11]. BC is synthesised mainly by extracellular metabolic pathways in non-photosynthetic bacteria like *Komagataeibacter nataicola* (TISTR 975) and *Acetobacter xylinum* [12], using glucose and basic hexose sugars, which are biosynthesised in a continuous 3D network of nano-fibrils at the liquid–air interface of the fermentation vats [10,13]. While *Acetobacter xylinus* has long been established as a reliable source for bacterial cellulose production, *Komagataeibacter nataicola* has become increasingly popular due to its innate resistance to ethanol and acetic acid, a common byproduct of cellulose production. In addition, *K. nitaicola* has higher adaptability to nutrient sources, allowing the use of sustainable carbon sources such as coconut water or corncob residues, which contain various sugars, amino acids, vitamins, and minerals [14,15]. Unlike plant cellulose, BC is naturally pure and contains no complex plant cellular fractions like hemicellulose, pectin, or toxic and immunogenic lignin; hence, the need for extensive purification by chemical methods is reduced. The natural purity and the structure of the nanofibrils make it easy to process in challenging medical applications, in which homogeneity and integrity of the material are critical. Moreover, the uniaxial orientation of these fibril bundles can be used to produce nanocomposites with improved tensile properties, as well as tailored surface functionalities [16]. However, the high mesh density may also have drawbacks for infiltration of cells, requiring the use of porogens or sophisticated manufacturing methods to achieve the best porosity for tissue regeneration [17,18].

Polyhydroxyalkanoates (PHAs) constitute a variety of completely biodegradable and biocompatible natural linear polyesters, which are accumulated as energy reserve inclusions in the cells of specific bacterial species under specific nutrient-limiting stress conditions. PHAs vary in monomer chain length and are either very rigid and brittle (SCL-PHA, 3–5 carbon atoms, known as poly-3-hydroxybutyrate or PHB) or very flexible like rubber (MCL-PHA, 6–14 carbon atoms) [19]. Most importantly, the PHA is degraded in vivo by fully controlled hydrolytic reaction, which results in innocuous organic metabolites (e.g., 3-hydroxybutyric acid) that are metabolically incorporated into living tissue matrices without any local inflammatory response [20].

Bacterial cellulose has been proven to be a potential material for Guided Tissue Regeneration (GTR) membranes in advanced periodontal therapy [21,22]. GTR membranes are applied in the treatment of severe periodontitis, a chronic inflammatory disease that causes the progressive loss of periodontal ligament and alveolar bone, which can result in tooth loss. In regenerative surgery, these hydrogel membranes serve as selective barriers that promote tissue regeneration by allowing for the slow migration of epithelial cells and connective tissue cells into the defect site while slowing the migration of periodontal ligament cells and osteogenic cells into the defect site to promote functional tissue regeneration [23,24]. The performance requirements for the GTR membranes are very demanding, such as high biocompatibility without cytotoxicity, adequate permeability for the exchange of nutrients and fluid, and mechanical stability to resist crushing during mastication, in addition to a carefully controlled biodegradation profile that matches the healing of tissues.

Life Cycle Assessment (LCA) is a very well-standardized method developed under the ISO 14040 and 14044 standards that systematically calculates the environmental impacts of a product over its entire life cycle [25,26]. The cradle-to-gate assessment approach is widely used to compare the environmental effects associated with the synthesis of new experimental biomaterials with the corresponding effects of existing commercial biomaterials [27]. This methodological background is of particular importance because conventional clinical requirements often involve the use of products derived from livestock when bovine collagen is used or petrochemicals such as ePTFE. These products are associated with enteric methane emissions and resource consumption due to the issuance of crops or feed, or concerns around long-term environmental persistence of petrochemical-based polymers, respectively. To overcome these difficulties, bacterial cellulose (BC) and its composites are developed, which can be synthesized using locally available agro-industrial wastes like mature coconut water [10,12,28]. The use of LCA in product development will allow researchers to assess critical sustainability factors, including global warming potential (GWP) and non-renewable energy use (NREU), and find environmental “hotspots” to ensure the sustainability of medical products [5,11,27].

This study emphasizes the necessity and significance of producing locally available, sustainable biomaterials with mechanical properties comparable to GTR membranes, such as bacterial cellulose. Using coconut water as a growth medium, this work sought to synthesise BC membranes from *Komagataeibacter nataicola* and enhance their physical characteristics by creating an in situ composite with PHA. The BC/PHA composite hydrogel is synthesised in situ by spreading PHA powder throughout the culture media. This exploratory study establishes the optimisation parameters for the fermentation process, the mechanics of in situ composite synthesis, and the systematic profiling of the physical and mechanical characteristics of this unique BC/PHA composite material for dental clinical translation potential.

## 2. Results and Discussion

### 2.1. Pre-Optimisation of Culture Conditions for Cellulose Production

Initial screening between the candidate bacterial strains conclusively revealed that only *Komagataeibacter nataicola* (TISTR 975) was capable of consistently self-assembling a uniform, structurally sound, continuous extracellular cellulose pellicle sheet under the specified coconut water boundaries. In contrast, *Komagataeibacter xylinus* (TISTR 1011) exhibited structural sedimentation, forming localised particulate aggregates that failed to fuse into a cohesive membrane sheet, eliminating it from subsequent optimisation loops. This is likely due to *K. xylinus* lack of specific metabolic pathways required to mitigate the inhibitory compounds often present in raw or improperly processed media, unlike *K. nataicola*, which exhibits robust adaptation to these complex ecological niches [14,15,29].

As shown in Table 1, the maximum wet weight reached 14.15 ± 0.06 g when utilising a sucrose concentration of 50 g/L over 9 days. In contrast, the maximum dry weight of the gel was recorded at 0.7553 ± 0.0006 g under a lower concentration of 45 g/L sucrose for 9 days. Structural measurements indicated that the peak wet thickness of approximately 2.42 mm (specifically 2.418 ± 0.407 mm) and the peak dry thickness of 0.1750 ± 0.0270 mm were both achieved at the 45 g/L sucrose and 9-day mark. These results demonstrate that while maximum hydration (wet weight) is supported by moderate carbon levels, the most significant fiber accumulation and overall membrane thickness are prioritised at slightly lower sugar concentrations over the full 9-day cultivation duration.

### 2.2. Fourier-Transform Infrared (FTIR) Spectroscopy Analysis

**Figure 1:**
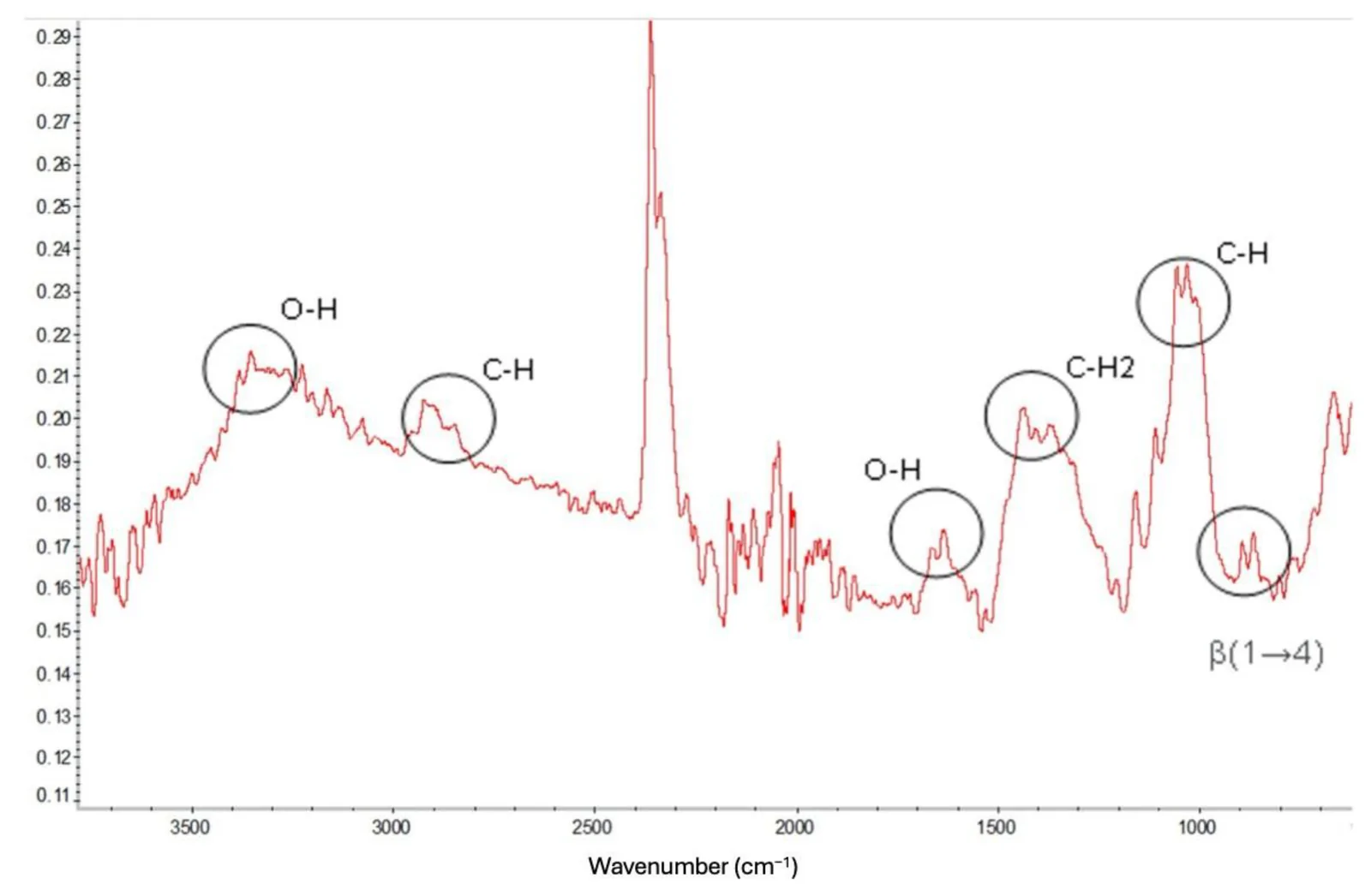
FTIR spectrum of bacterial cellulose produced by *Komagataeibacter nataicola* (TISTR 975).

The chemical architecture and macromolecular functional groups of the optimised pure bacterial cellulose were mapped using FTIR spectroscopy across a wavenumber range of 4000 to 400 cm^−1^ as shown in Figure 1. The resulting FTIR spectrum for the pure BC hydrogel exhibited the classic signatures of a highly crystalline cellulose polymorph. A strong, broad absorption band centered at 3340 cm^−1^ was observed, corresponding to the stretching vibrations of the hydroxyl groups (O–H) involved in extensive intra- and intermolecular hydrogen bonding networks [30]. The sharp peak at 2890 cm^−1^ is attributed to the asymmetric C–H stretching vibrations of aliphatic CH_2_ groups in the glucose monomer units. Furthermore, prominent absorption bands emerged at 1160 cm^−1^ and 1050 cm^−1^, which are characteristic of asymmetric C–O–C bridge stretching and C–O skeletal vibrations of the glucan rings, respectively. The absence of any absorption peaks around 1700–1750 cm^−1^ verified that the purified baseline material was entirely composed of pure cellulose, confirming that the post-harvest sodium hydroxide (NaOH) washing protocol successfully eliminated all residual bacterial proteins, nucleic acids, and media components without disrupting the polysaccharide structural framework.

### 2.3. Water Content and Swelling Ratio

A critical performance metric for an effective GTR dental membrane is its fluid absorption capacity, which governs the gel’s ability to absorb blood clots, retain physiological fluids, and mediate nutrient/growth factor transport at the surgical defect site. The fluid dynamics of the optimised pure dried BC membrane were tracked via its equilibrium water content (EWC) over a 60-min immersion period.

**Figure 2:**
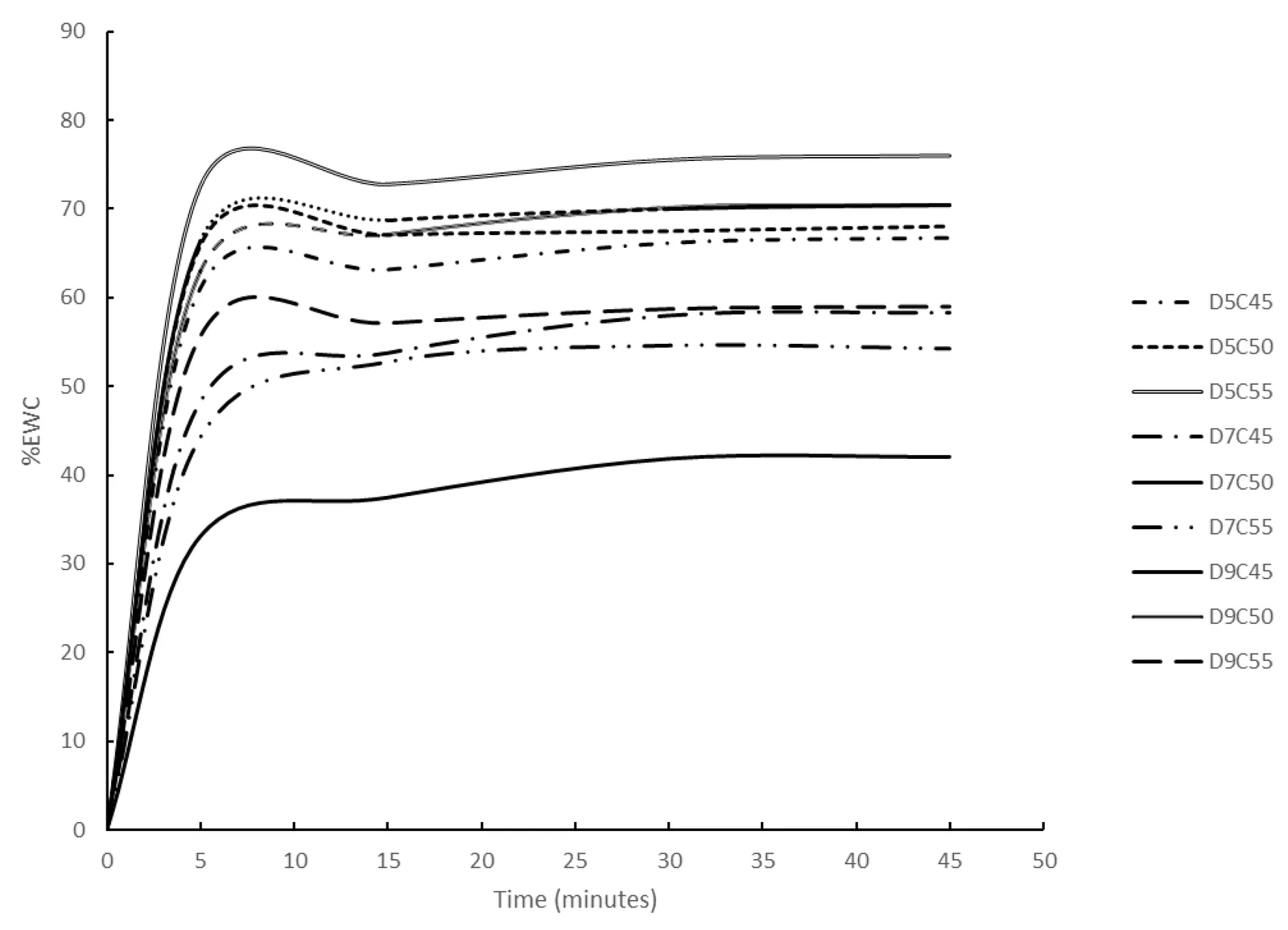
Equilibrium water content (EWC) of pure bacterial cellulose membrane (day of culture 5, 7, and 9 with carbon source 45, 50, and 55 g/L).

The pure BC film demonstrated ultra-rapid capillary fluid intake during the initial phases of rehydration. Within the first 5 to 10 min of submersion in deionised water, the membrane experienced a massive swelling burst as seen in Figure 2. This rapid swelling behaviour is likely due to the highly hydrophilic nature of the exposed hydroxyl groups on the nanofibrillar surfaces, combined with the microscopic porous voids within the dehydrated three-dimensional network that generate strong capillary forces.

As the immersion time progressed past 30 min, the fluid absorption rate plateaued, indicating that the internal interstitial spaces of the cellulose grid had reached volumetric saturation. The highest stable EWC achieved was with the 55 g/L carbon source, over 5 days of incubation, and the lowest EWC ratio at approximately 40% was reached with the 45 g/L carbon source, over 9 days of incubation.

### 2.4. Mechanical Performance Evaluation of Pure Cellulose Membrane

**Figure 3:**
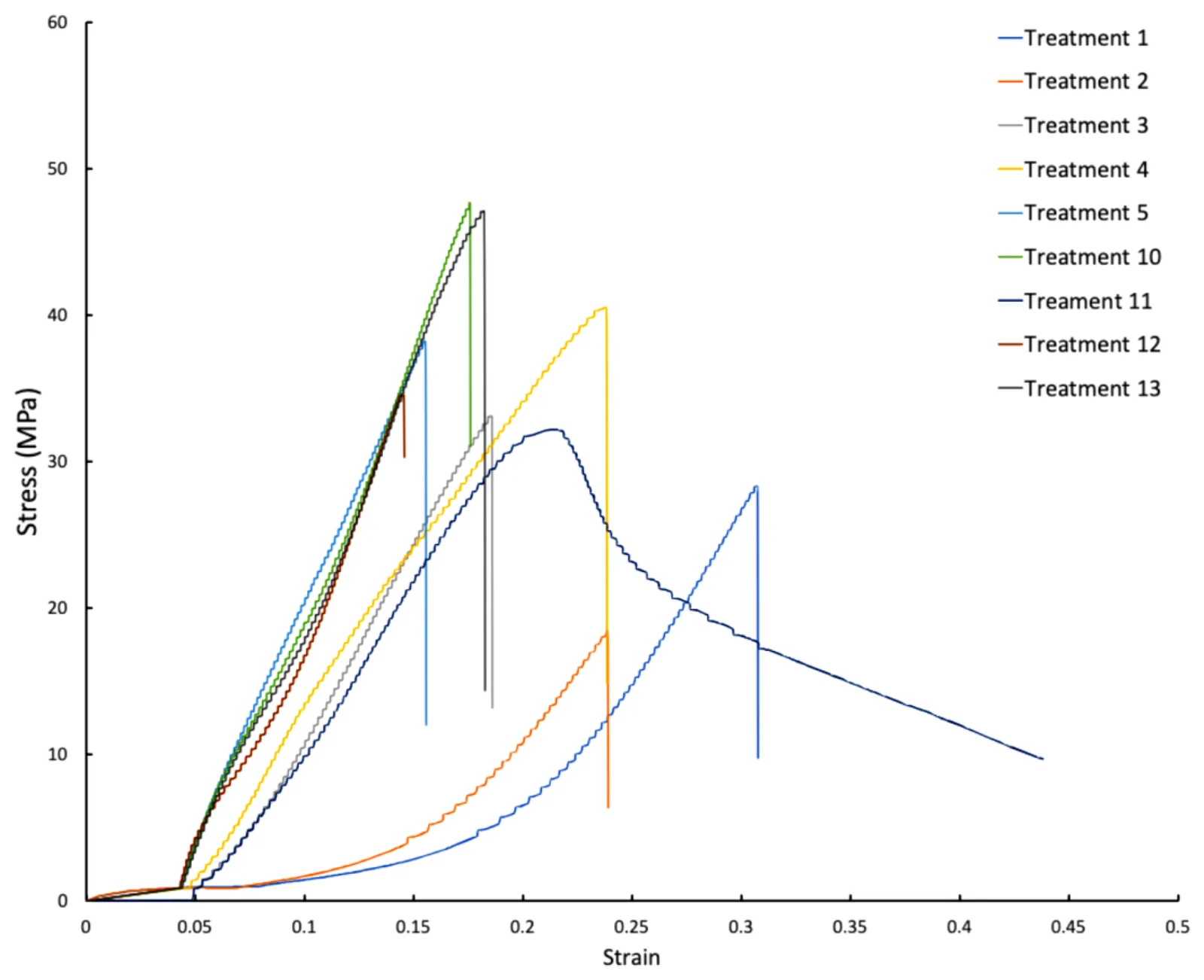
Stress/strain curves of all tested pure bacterial cellulose membranes; Treatment 1–3: 5 days of bioproduction of BC with 45, 50 and 55 g/L carbon source. Treatments 4, 5 and 10: 7 days of bioproduction of BC with 45, 50 and 55 g/L carbon source. Treatments 11–13: 9 days of bioproduction of BC with 45, 50 and 55 g/L carbon source, respectively.

Mechanical integrity is essential for GTR applications to prevent the membrane from collapsing into the focal bone defect under the pressure of overlying gingival tissue and masticatory forces. Uniaxial tensile testing was executed on the dried pure BC strips to define their structural boundaries. The definitive mechanical characteristics of each membrane condition are shown in Table 2 and Figure 3. Overall, the stress–strain curves demonstrate a clear trade-off between stiffness and ductility across the different groups. While most treatments exhibit similar high stiffness but brittle characteristics, treatments 1 and 2 display relatively lower stiffness but increased mechanical compliance and ductility indicated by higher strain values. Notably, treatment 11 exhibits non-linear behavior after its peak stress of approximately 32 MPa, showing a gradual, ductile decline in stress before failure at a higher strain (~0.44), suggesting potential plastic deformation capacity.

The stress–strain configuration revealed that uncomposited pure bacterial cellulose acts as a highly crystalline, rigid polymer. The BC membrane from 45 g/L carbon source over 9 days of incubation demonstrated the highest Ultimate Tensile Strength (UTS) of 49.87 ± 2.39 MPa. This exceptional load-bearing capability is directly attributed to the dense, highly ordered arrangement of microfibrils and the dense network of intermolecular hydrogen bonds established during the optimised 7-day bioprocess of bacterial cellulose. However, this high tensile rigidity came at the expense of material flexibility, as evidenced by a restricted elongation at break of 17.83 ± 0.67% and a steep Young’s Modulus of 334.62 ± 24.04 MPa. The mechanical profiling suggests that while pure BC hydrogel possesses the necessary baseline structural strength to resist anatomical compression, its highly rigid and relatively brittle nature presents practical handling limitations. Without modifying its elasticity, the pure BC gel risks micro-fracturing or tearing when subjected to complex surgical contouring, adapting to curved alveolar ridges, or under structural stress from clinical suturing.

### 2.5. Cellulose Optimisation via Response Surface Methodology (RSM)

To systematically evaluate and optimise the extracellular synthesis kinetics of baseline bacterial cellulose (BC) by *Komagataeibacter nataicola* (TISTR 975), a face-centered central composite design (CCD) under Response Surface Methodology (RSM) was executed as demonstrated in Table 3. Two independent variables—exogenous sucrose loading concentration (Xa: 45, 50, and 55 g/L) and static fermentation duration (Xb: 5, 7, and 9 days)—were each examined at three levels and evaluated against critical structural and mechanical responses: wet membrane mass (Y1, g), synthesised film thickness (Y2, mm), and structural Young’s modulus (Y3, MPa). The design comprised 13 experimental runs, including five replicated center points (50 g/L, 7 days), to estimate experimental error and assess model reproducibility.

The relationships between the two independent variables and each response were expressed by the following second-order polynomial regression equations (in actual units), where Xa denotes sucrose concentration (g/L) and Xb denotes cultivation period (days):

$$Wet\;Mass\left(g\right)Y_{1}=85.4-4.66\;Xa+8.51\;Xb+0.0445\;{Xa}^{2}-0.641\;{Xb}^{2}+0.0520\;Xa\;Xb$$

$$Wet\;Thickness(mm)=30.68-1.211\;Xa-0.107\;Xb+0.01195\;{Xa}^{2}+0.0208\;{Xb}^{2}+0.00291\;Xa\;Xb$$

$$Young's\;Modulus\;\left(MPa\right)Y_{3}=1129-105.1\;Xa+402\;Xb+1.117\;{Xa}^{2}-25.13\;{Xb}^{2}$$

**Figure 4:**
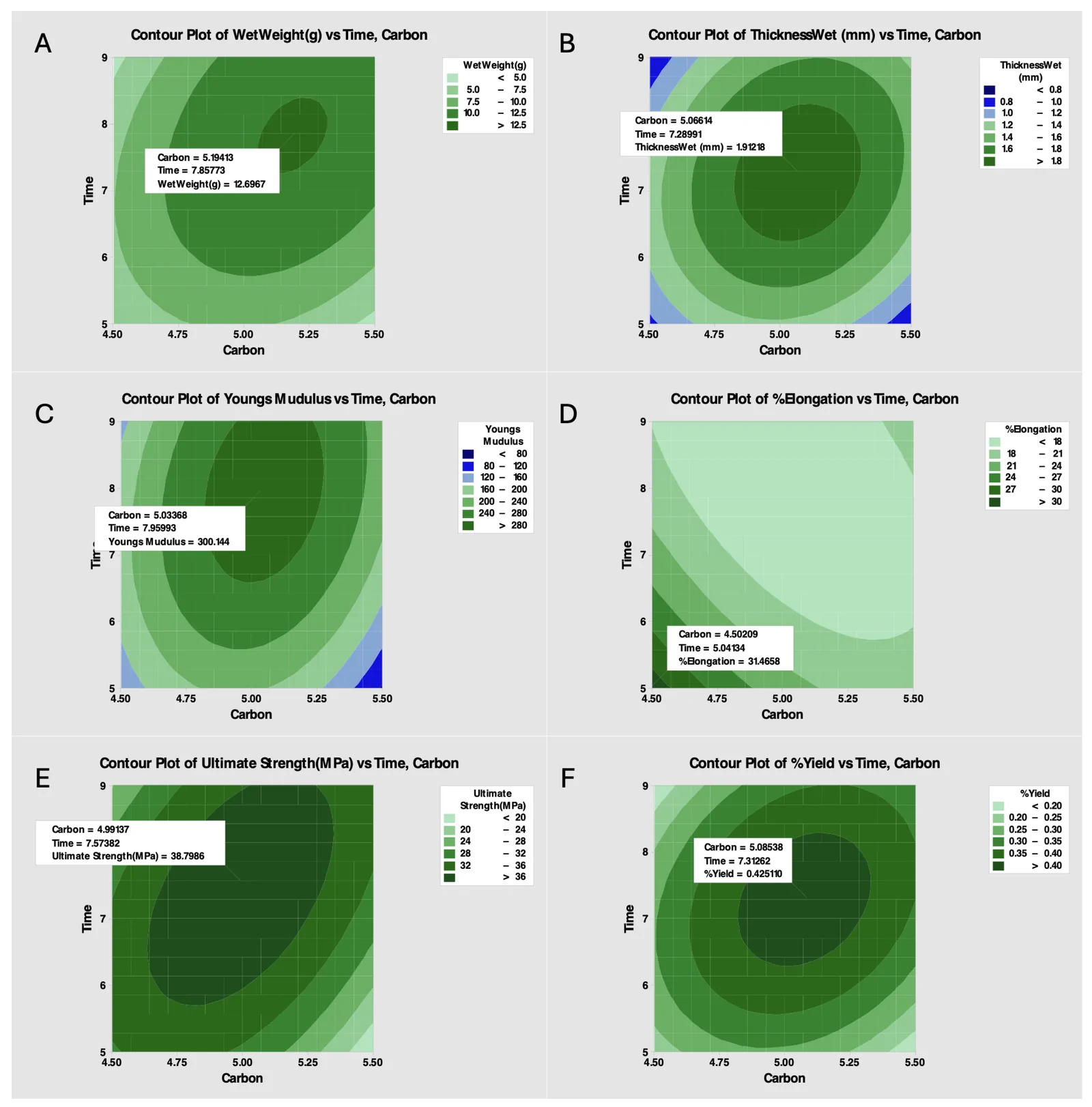
The contour plot representing the response surface for each variable vs. time, carbon: (**A**) wet membrane thickness, (**B**) wet weight, (**C**) Young’s modulus, (**D**) elongation, (**E**) ultimate tensile strength, and (**F**) yield.

The RSM analysis of physical yield depicted in Figure 4 demonstrated that both sucrose availability and time significantly influenced fiber accumulation. The maximum wet weight (14.15 ± 0.06 g) was observed when using a sucrose concentration of 50 g/L over 9 days, indicating that moderate sugar levels over longer durations maximise the material’s water-holding capacity. Conversely, the maximum dry weight (0.7553 ± 0.0006 g) and the highest yield percentage (0.7553%) were achieved at a lower sucrose concentration of 45 g/L after 9 days. This divergence suggests that while higher sugar concentrations might increase the total volume of the hydrated pellicle, slightly lower concentrations may promote a denser cellulose network within that volume.

In terms of structural characteristics, the thickness of the BC hydrogel was found to be highly responsive to both variables. While the maximum wet thickness (~2.42 mm) occurred at the 9-day mark for both 45 g/L and 55 g/L concentrations, the researchers prioritised thickness homogeneity—a consistent profile across the entire sheet—which was most reliably produced at the 50 g/L sucrose and 7-day mark. This consistency is critical for dental barrier membranes, as any significant variation in thickness could lead to structural failure or a breach in the barrier function during the tissue regeneration process. The mechanical response surface provided the most vital data for clinical application, showing clear trade-offs between strength and elasticity. The Ultimate Tensile Strength peaked at 49.87 ± 2.39 MPa under the maximum levels of 55 g/L sucrose and 9 days of growth, indicating that extended nutrient exposure leads to a harder material. However, the maximum elasticity (%Elongation) of 36.71 ± 8.50% was recorded during the early growth stage (5 days) with 45 g/L of sucrose, as the network becomes increasingly rigid and less flexible as fibre density increases over time. The peak Young’s Modulus, which measures the material’s stiffness and resistance to deformation, was maximised at 50 g/L sucrose and 7-day condition (323.94 ± 65.06 MPa). By employing multi-response desirability optimisation profiles, the statistical model localised the absolute processing optimum at an additional sucrose concentration of 50 g/L and a cultivation period of exactly 7 days. Although some conditions offered higher ultimate strength or absolute weight, this precise matrix configuration provides a balance of biological efficiency with structural specifications, producing a highly uniform membrane baseline possessing a reliable wet yield (9.85 ± 0.06 g), a stable cross-sectional thickness (1.22 ± 0.10 mm wet; 0.057 ± 0.005 mm dried), and a highly rigid baseline stiffness (323.94 ± 65.05 MPa). Thus, this condition was selected for further testing. The predictive accuracy and reliability of the model were demonstrated through a comparison of experimental results with model-generated values (Table 4), yielding high coefficients of determination for primary structural and mechanical metrics: 0.9451 for wet thickness, 0.9483 for ultimate tensile strength, 0.8927 for Young’s Modulus, and 0.8881 for dry weight. These values indicate that over 94% of the variance in membrane thickness and strength is effectively explained by the optimised cultivation parameters, confirming the robustness of the statistical design. Further analysis of the maximum prediction error revealed high precision for critical performance indicators, with a maximum absolute error of only 3.53 MPa for ultimate tensile strength and 0.23 mm for wet thickness. While the maximum prediction error for Young’s Modulus was calculated at 67.81 MPa, this figure remains within one standard deviation of the experimental mean, verifying that the model accurately captures the overall trend of material stiffness. Additionally, the maximum prediction errors for wet weight (2.45 g), dry weight (0.12 g), and dry thickness (0.04 mm) were documented, collectively demonstrating that the identification of 50 g/L sucrose and a 7-day cultivation period as optimal is statistically sound for producing uniform bacterial cellulose membranes.

### 2.6. Composite Cellulose Comparison

**Figure 5:**
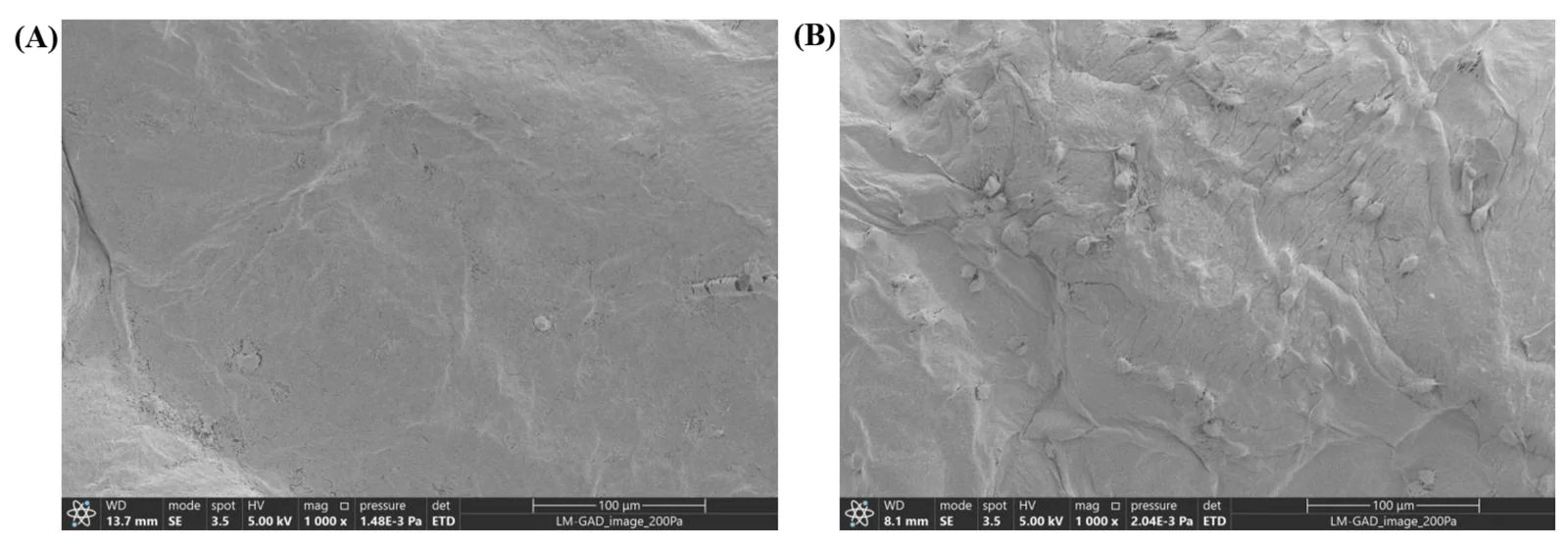
SEM micrographs (1000×) comparing the surface morphology of (**A**) the pure bacterial cellulose membrane and (**B**) the bacterial cellulose/PHA composite membrane. The pure BC membrane shows a smooth, folded cellulose surface with no particulate features, whereas the composite membrane exhibits discrete PHA particles distributed across the membrane surface.

The surface microstructure of the pure BC and BC/PHA composite membranes was examined by SEM at 1000× magnification (Figure 5). The pure BC membrane exhibited a smooth, homogeneous surface with a folded, sheet-like texture and no discrete particulate features. The absence of clearly resolved individual fibrils is attributed to the drying and compaction of the pellicle, during which the hydrated three-dimensional nanofibril network collapses into a densely packed, film-like layer rather than remaining as discrete, separated fibrils, as is typically observed in wet BC pellicles. In contrast, the BC/PHA composite membrane displayed numerous discrete particulate features distributed across the membrane surface, corresponding to the PHA introduced during the in-situ synthesis. These particles were polydisperse, with observed dimensions ranging from approximately 5 to 30 µm.

These micrographs provide direct evidence of the successful in situ incorporation of PHA into the bacterial cellulose network. Notably, the particles observed in the composite are substantially larger than the nominal 1 µm particle size of the PHB powder as supplied [31]. This indicates that agglomeration occurred during dispersion in the culture medium and over the course of the seven-day static fermentation, most plausibly as a consequence of the hydrophobic character of PHA, which favours particle–particle association in the aqueous medium over particle–cellulose association. Importantly, the particles persist as discrete, well-defined entities rather than being dissolved or assimilated, confirming that they remain a distinct phase throughout the cultivation period. Taken together, these observations indicate that the interaction between the hydrophobic PHA and the hydrophilic cellulose is physical rather than chemical in nature: the PHA aggregates are physically entrapped within the cellulose network as it self-assembles around them, acting as discrete inclusions rather than forming covalent or hydrogen-bonded linkages with the cellulose chains. Despite this agglomeration, the particles are distributed across the membrane surface rather than being confined to localised regions, indicating that the in situ dispersion approach achieves incorporation throughout the membrane.

**Figure 6:**
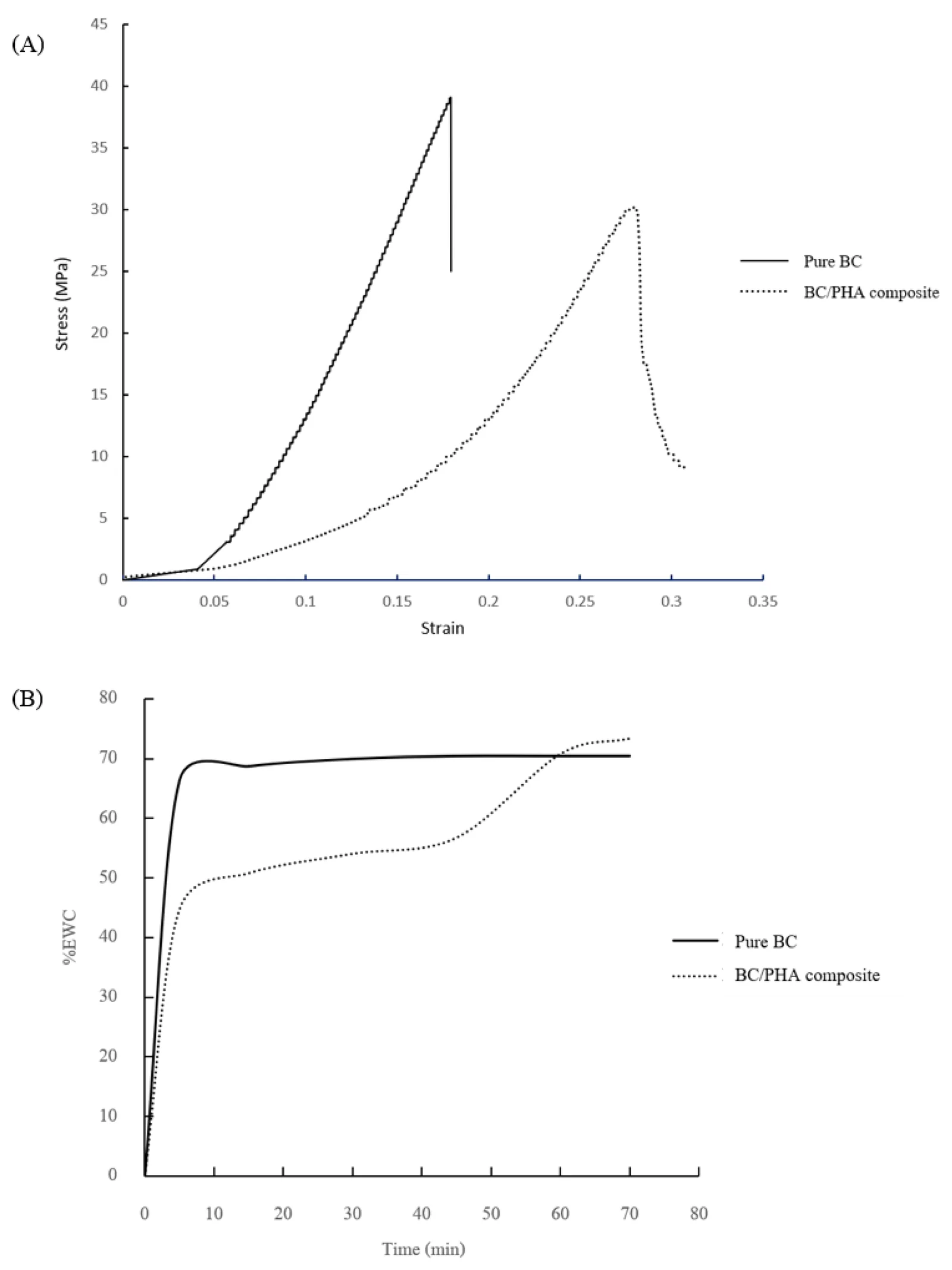
A comparison between pure bacterial cellulose membrane vs. bacterial cellulose/PHA composite membrane. (**A**) Ultimate tensile strength of pure BC vs. BC/PHA composite. (**B**) Equilibrium water content of pure BC vs. BC/PHA composite.

The comparative characterisation of pure bacterial cellulose (BC) and bacterial cellulose/polyhydroxyalkanoate (BC/PHA) composite hydrogel membranes reveal distinct mechanical and physical performance profiles. As shown in Figure 6, pure BC synthesised under optimal conditions exhibits a superior ultimate tensile strength of 37.67 ± 3.11 MPa and greater stiffness, represented by a Young’s Modulus of 323.94 ± 65.06 MPa, whereas the BC/PHA composite presents a lower strength of 33.76 ± 3.60 MPa and a reduced modulus of 177.51 ± 65.33 MPa. From a clinical handling perspective, this increased flexibility and elasticity are critical advantages for Guided Tissue Regeneration (GTR) membranes. Pure BC membranes are characterised by high stiffness, which may lead to brittleness or difficulty in adapting the membrane to the complex, contoured surfaces of the oral cavity. The lower Young’s Modulus of the BC/PHA composite results in a more pliable material that is easier for a surgeon to manipulate and secure during dental procedures. Regarding the ability to resist typical oral forces, the synthesised BC/PHA composite remains highly robust when compared to established clinical benchmarks [32]. Even with the slight reduction in strength caused by PHA integration, the experimental composite’s UTS of 33.76 MPa is approximately 2.5 to 7 times higher than these widely used commercial alternatives (Table 5). This indicates that the membrane possesses a substantial safety margin to successfully resist masticatory loads and other intraoral mechanical stresses.

In addition, the in situ integration of 0.1% wt. PHA powder significantly enhances material ductility, resulting in an elongation at break of 28.20 ± 0.05% for the composite compared to 22.91 ± 0.05% for the pure cellulose pellicle. Fluid interaction studies further differentiate the two materials, showing that while pure BC achieves rapid saturation within 10 min at a water equilibrium of approximately 70.93 ± 1.57%, the BC/PHA composite reaches a higher total equilibrium water content of 73.56 ± 1.82% over a longer duration of approximately 60 to 70 min.

The microstructural features revealed in Figure 5 provide a mechanistic basis for the mechanical and fluid-handling behaviour described above. The PHA inclusions entrapped within the cellulose network are expected to locally interrupt the continuity of the dense inter-fibrillar hydrogen-bonding network that confers rigidity on pure BC. This interruption increases the effective spacing between cellulose fibres and permits greater fibre slippage under tensile loading, which accounts for the marked increase in elongation at break observed for the composite. The same discontinuity, however, disrupts the efficient transfer of load through the continuous cellulose network and introduces sites of stress concentration at the particle–matrix interface, an effect that is likely accentuated by the agglomerated nature of the inclusions, and which explains the concomitant reduction in ultimate tensile strength and Young’s modulus. The trade-off between strength and ductility observed here is therefore a direct structural consequence of the physical, rather than chemical, mode of PHA incorporation. The fluid-uptake behaviour can be interpreted along the same lines: the increased inter-fibre spacing enlarges the free volume available within the three-dimensional network and thereby raises the total equilibrium water content of the composite, while the hydrophobic nature of the dispersed PHA particles retards capillary infiltration, accounting for the slower but ultimately more voluminous absorption relative to pure BC. Consequently, the composite membrane demonstrates improved mechanical elasticity and total water-holding capacity relative to pure bacterial cellulose, at the cost of only a modest reduction in peak strength.

**Figure 7:**
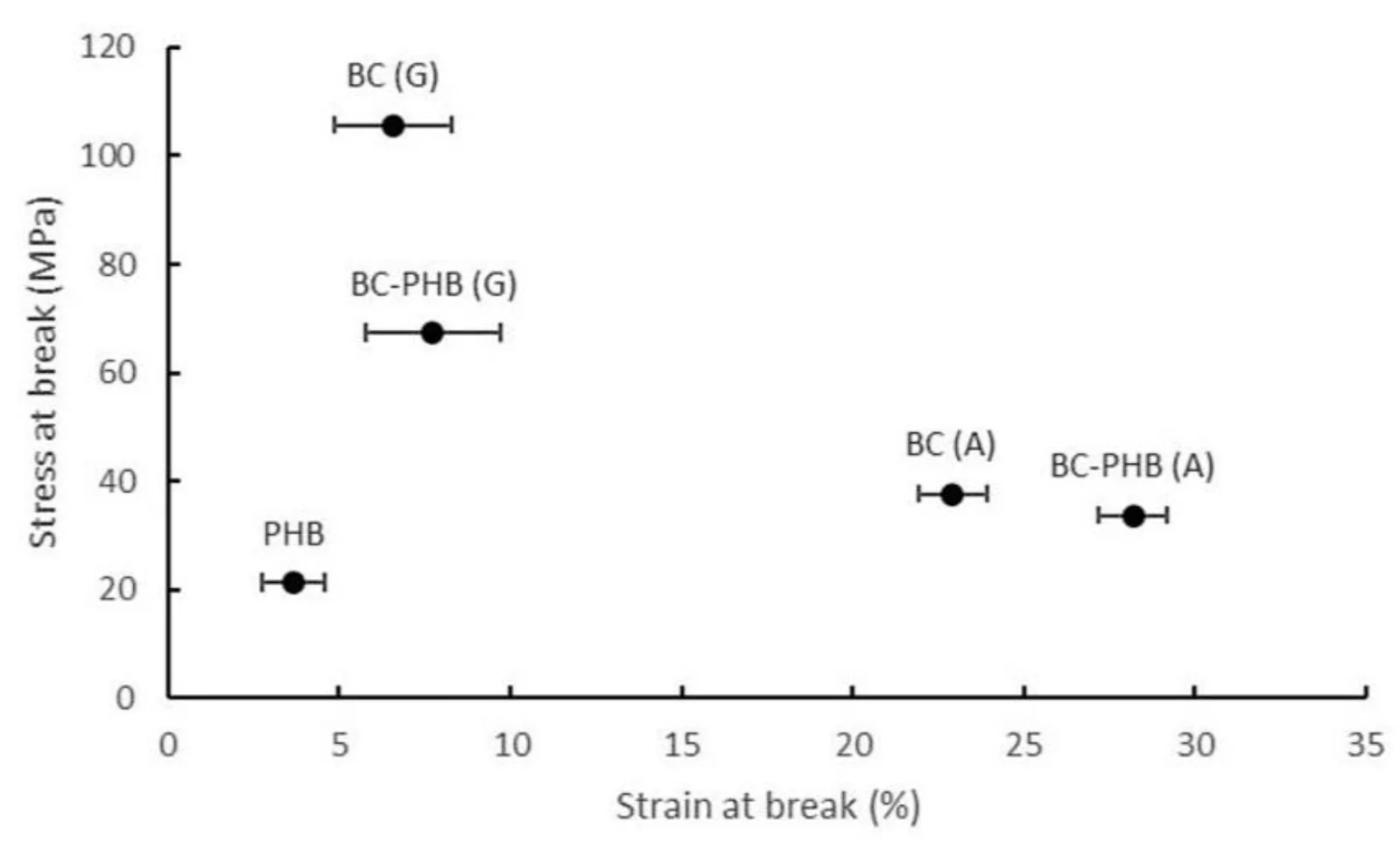
Mechanical properties comparison between this study and existing publication. BC(A): *Acetobacter xylinum* (ATCC 23767) cellulose; BC(G): *Gluconacetobacter xylinus* (ATCC 53524) cellulose; PHB: Poly-3-hydroxybutyrate; BC-PHB(A): *Acetobacter xylinum*–PHB composite; BC-PHB(G): *Gluconacetobacter xylinus*–PHB composite.

In comparison to existing previous research work [33], while the BC hydrogel synthesised in this study from *Acetobacter xylinum* (ATCC 23767) demonstrated a markedly higher elongation at break (22.91%) compared to the *Gluconacetobacter xylinus* (ATCC 53524) reported in previous publications (6.57%), the latter exhibited a superior ultimate tensile strength of 105.66 MPa compared to the 37.67 MPa achieved in this research (Figure 7). Similarly, the BC-PHB gel composite developed in this work reached an elongation of 28.20%, which is significantly higher than the 7.74% reported for comparable composites in earlier studies [33]. Moreover, the thickness and elasticity of the synthesised BC/PHA composite were similar to the accepted commercial collagen membranes (Sigma-Aldrich), which are qualified in separate mechanical evaluations of dental materials. Results are in line with the reported literature, indicating that in situ polymerisation of polymers such as PHAs or PHB increases the fluid absorption properties and ductility of the hydrogel at the cost of its peak tensile modulus and thus provides a biomaterial with optimum material properties for medical applications.

### 2.7. Life Cycle Assessment (LCA)

**Figure 8:**
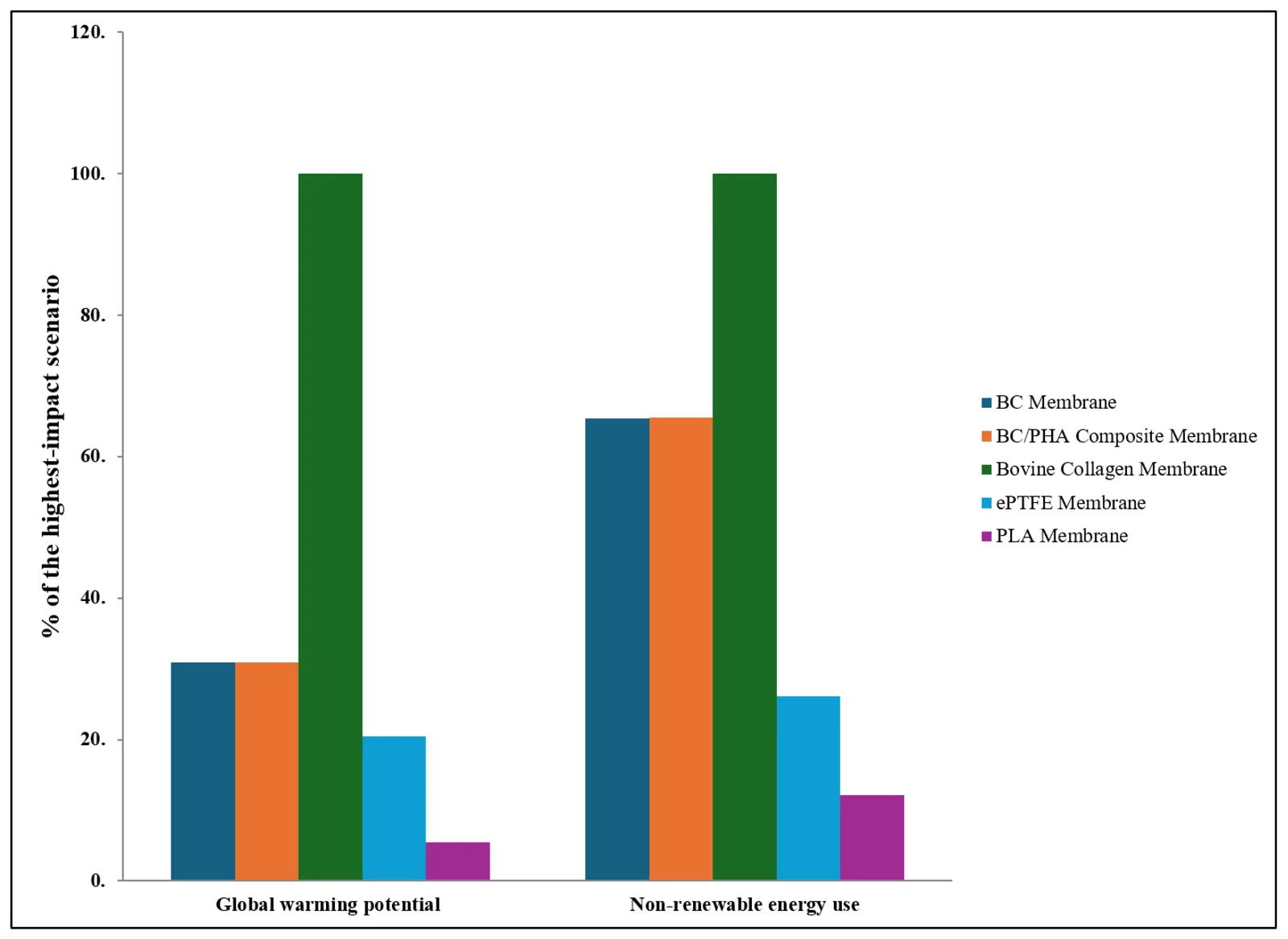
Comparative cradle-to-gate environmental impacts of the five guided tissue regeneration (GTR) membrane scenarios, pure BC, BC/PHA composite, bovine collagen, ePTFE, and PLA for global warming potential (climate change, short term) and fossil and nuclear energy use, assessed per functional unit (one 15 × 25 mm membrane) using the IMPACT World+ Midpoint method. Results are expressed relative to the highest-impact scenario (set to 100%) within each category.

The cradle-to-gate global warming potential (GWP) and non-renewable energy use (NREU) reported through the fossil and nuclear energy use indicator of the five GTR membrane scenarios are summarised in Table 6 and visually depicted as relative impacts in Figure 8. For both indicators, the scenarios ranked from lowest to highest environmental burden as follows: PLA, ePTFE, pure BC, BC/PHA, and bovine collagen.

The bovine collagen membrane exhibited by far the greatest impact. As illustrated in Figure 8 (set to 100% for the highest-impact scenario), it exceeded the experimental BC/PHA membrane by approximately 3.2-fold in GWP and 1.5-fold in NREU. Contribution analysis attributed roughly 57% of the bovine GWP directly to live-weight cattle rearing rather than to downstream membrane processing. This is consistent with the well-documented environmental intensity of livestock-derived inputs, in which enteric methane and feed cultivation dominate the upstream inventory [34].

The pure BC and BC/PHA membranes returned nearly identical GWP and NREU values. This indicates that the in situ incorporation of PHA microspheres (0.005 g per functional unit) introduces a negligible additional environmental burden, while crucially delivering the necessary mechanical improvements reported in Section 2.6. The principal hotspot for both BC scenarios across both indicators was the electricity consumed during the 7-day static fermentation and sterilisation processes. This electricity is drawn from the Thai national grid, which remains heavily reliant on natural gas and coal. However, because this burden originates from processing electricity rather than from the feedstock itself, it is intrinsically scalable [35].

Notably, at the laboratory scale assessed here, both BC membranes returned higher GWP and NREU than the synthetic ePTFE and PLA comparators. This reflects the energy intensity of bench-scale fermentation relative to the minimal processing energy embodied in a small mass (0.06 g) of pre-manufactured polymer resin, rather than an inherent disadvantage of bacterial cellulose. Two pathways are therefore expected to narrow this gap substantially: industrial-scale production, which raises the energy yield per membrane through full-capacity batch utilisation, and the progressive decarbonisation of the national electricity supply. A sensitivity analysis based on maximum equipment loading, allocating incubator, autoclave, and lyophiliser capacity according to the membrane footprint, confirmed that per-unit electricity, and therefore both the GWP and NREU of the BC scenarios, scales inversely with batch size.

A direct numerical comparison across all five membranes is, however, potentially misleading because the scenarios represent two clinically distinct functional classes. The ePTFE membrane is non-resorbable and mandates a second surgical procedure for removal once tissue regeneration is complete. In contrast, the BC/PHA, collagen, and PLA membranes are resorbable and degrade in situ, eliminating this clinical requirement. Consequently, the comparatively low cradle-to-gate GWP and NREU of ePTFE do not translate into clinical or environmental superiority; the chosen system boundary excludes the burdens associated with the second intervention as well as the long-term persistence of a non-degradable, PFAS-class fluoropolymer [36].

A more equitable interpretation therefore compares membranes within their functional class. Among the resorbable alternatives, the experimental BC/PHA hydrogel substantially outperformed the bovine collagen baseline while remaining within the same order of magnitude as PLA. This positions it as a highly competitive resorbable option that simultaneously avoids livestock-associated impacts and fluorochemical persistence.

It must further be emphasised that the functional unit adopted here defines geometric and mass equivalence, a structurally intact 15 × 25 mm membrane of comparable dry weight but does not encode equivalence in clinical performance. Key in-service properties governing therapeutic effectiveness, including fluid absorption capacity, swelling behaviour, degradation kinetics, and mechanical resilience under masticatory load, differ substantially among the five materials and cannot be normalised within the inventory framework. The GWP and NREU values reported here, therefore, quantify the environmental burden of producing one physical membrane unit, not the burden per unit of delivered clinical function. The present results should accordingly be weighed alongside the physicochemical performance data established in Section 2.3, Section 2.4, Section 2.5 and Section 2.6 rather than read as a standalone ranking. For the BC/PHA hydrogel specifically, its competitive production footprint, combined with the stable equilibrium water content and flexibility demonstrated in this work, indicates a favourable balance between environmental cost and functional capability that production-only indicators cannot fully capture [37].

Beyond the quantified indicators, the BC/PHA membrane offers qualitative sustainability advantages aligned with the socioeconomic motivation established earlier in this study. It is derived from mature coconut water, an agro-industrial residue valorised here as a burden-free feedstock under the cut-off allocation approach. This draws on a locally abundant by-product rather than a dedicated petrochemical or livestock supply chain. A resorbable, fluorine-free, locally producible BC/PHA membrane whose dominant burden is a single, scalable, and decarbonisable process input, therefore, represents a credible pathway toward more sustainable and accessible guided tissue regeneration therapy in Thailand.

## 3. Conclusions

This study aimed to optimise the production of bacterial cellulose (BC) from *Komagataeibacter nataicola* TISTR 975 using mature coconut water as a sustainable fermentation medium. The optimum medium to produce uniform, stiff membranes was determined by response surface methodology, yielding 50 g/L sucrose and a 7-day cultivation period, and was confirmed by the strain screening. *K. nataicola* TISTR 975 was found to be suitable to produce a continuous and cohesive pellicle in this medium, instead of *K. xylinus* TISTR 1011. Pure BC exhibited significantly higher tensile strength, but was brittle, had poor water-holding capacity and was difficult to process, so an in situ BC/PHA composite was prepared that had a slightly lower tensile strength and showed a more balanced combination of tensile strength, water-holding capacity, and ductility for membrane applications while closely matching the mechanical properties of commercially available collagen membranes. Chemical analysis of the produced cellulose confirmed its purity and its remarkable potential for being used in the field of guided tissue regeneration as a biodegradable membrane. A cradle-to-gate life cycle assessment also revealed that the global warming potential of the BC/PHA composite hydrogel was approximately 3.2 times lower than that of bovine collagen membranes, with the electricity consumed in static fermentation and downstream processing processes being the most significant impacts on the environment and PHA being a negligible contributor.

The novelty of this work lies in combining mature coconut water as a low-cost substrate, *K. nataicola* TISTR 975 as an effective production strain, and in situ BC/PHA composite formation to achieve improved mechanical performance and lower environmental impact. Future work should extend beyond basic cytocompatibility testing to include detailed cell adhesion, proliferation, and differentiation studies to confirm the membrane’s ability to support tissue regeneration. Barrier function should also be evaluated under conditions that better mimic the oral environment, including fluid challenge, mechanical loading, and long-term exposure to enzymes and physiological media. In addition, degradation behaviour should be examined more systematically to determine whether the hydrogel maintains structural integrity long enough to support healing before resorbing at an appropriate rate. From a translational perspective, larger-scale production studies are needed to verify reproducibility, process stability, and cost efficiency under industrial conditions. Finally, in vivo studies will be essential to assess tissue integration, host response, and regeneration outcomes before the material can be considered for clinical use.

## 4. Materials and Methods

### 4.1. Microbial Strain and Media Preparation

The baseline synthesis of raw bacterial cellulose was executed and comparatively evaluated using two primary target microorganisms obtained from the Thailand Institute of Scientific and Technological Research (TISTR): *Komagataeibacter nataicola* (TISTR 975) and *Komagataeibacter xylinus* (TISTR 1011). The primary liquid fermentation culture medium was derived using natural, mature coconut water sourced from domestic processing hubs, containing an initial natural sugar profile of 5–6° Brix. The default baseline production medium was prepared using the following precise configuration: 1 L of filtered mature coconut water, supplemented with 3.0 g/L of analytical-grade ammonium phosphate (NH_4_)_2_HPO_4_ (Sigma-Aldrich, St. Louis, MO, USA) as the essential inorganic nitrogen source, and 10.0 mL/L of glacial acetic acid to strictly regulate the initial culture pH to a highly favourable acidic window of 4.5–5.0, suppressing competitor microbial contaminants. The solution was sterilised in an autoclave system at 121 °C under 15 psi pressure for 20 min before inoculating the microbes. The bioproduction of bacterial cellulose was done under static conditions in a standard 500 mL glass beaker at 25–37 °C; 60% of the container was maintained as free headspace to ensure sufficient aeration. The starting inoculum represented 10–20% of the liquid media with a total culture volume of 200 mL.

The fabrication of the BC/PHA composite was achieved via an in situ methodology, where the secondary polymer was integrated during the biological growth of the cellulose. Polyhydroxyalkanoates (PHAs), specifically 0.1% wt. Poly-3-hydroxybutyric acid (PHB) powder (Sigma-Aldrich, St. Louis, MO, USA), were added directly into the culture medium after 3 days of cultivation. The particle size distribution analysis was performed in a previous study, and the approximate particle size was 1 µm. As the *K. nataicola* synthesised the 3D nanofibrillar structure, the PHA particles became entrapped and integrated within the gel matrix, resulting in a composite material with enhanced mechanical elasticity and fluid absorption properties compared to pure bacterial cellulose.

### 4.2. Optimisation via Response Surface Methodology

To establish the mathematical optimum for cellulose pellicle thickness, uniformity, and mechanical performance, a response surface methodology (RSM) based on a face-centered central composite design (CCD) was performed. Two continuous factors were each evaluated at three levels: (1) supplementary sucrose concentration at 45, 50, and 55 g/L; and (2) cultivation duration at 5, 7, and 9 days. In the face-centred configuration, the axial points were positioned on the faces of the design space (α = 1), such that all factor levels coincided with the low, centre, and high settings. This generated a matrix of 13 experimental fermentation runs, of which runs 5 through 9 were replicated centre points (50 g/L sucrose, 7 days) used to estimate experimental error and assess model reproducibility. Cultivations were maintained under static conditions in an incubator set to a temperature range of 25–37 °C.

### 4.3. Membrane Production

All fabricated membranes, whether pure or composite, underwent a rigorous purification protocol to remove residual bacterial cells and culture media components. The pellicles were first boiled in deionised water for 60 min, followed by immersion in a 0.1 M Sodium Hydroxide (NaOH) solution (Sigma-Aldrich, St. Louis, MO, USA) at 80 °C for 40 min. The purified hydrogels were then washed repeatedly with deionised water until reaching a neutral pH and were air-dried at a room temperature of 25 °C for three days to obtain a stable, dry membrane suitable for characterisation and performance testing.

### 4.4. Equilibrium Water Content (EWC)

The assessment of the liquid absorption capacity of the synthesised membranes was conducted by determining the Equilibrium Water Content (EWC) through a gravimetric analysis of the specimens. To ensure consistency, the membranes were cut into uniform dimensions of 10 × 10 mm and weighed to establish an initial dry mass baseline. These specimens were then immersed in 3 mL of deionised water at a controlled temperature of 25 °C, with each test performed in triplicate to maintain statistical reliability. The samples were removed from the fluid at specific time intervals—typically 5, 15, 30, and 45 min—to measure the weight gain after excess surface moisture was gently blotted away with filter paper. The percentage of water content was calculated as the weight gain relative to the final swollen weight of the material, following Equation (1):

(1) $$\%EWC=\frac{Ws\;-W_{0}\;\;}{Ws\;}\times 100$$

Ws = weight of the swollen polymer at a given time.

$W_{0}\;$= initial weight of the dry polymer.

The total absorption capacity was finally determined by plotting these percentage values against time until the hydration level stabilised, signifying that the biopolymer network had reached its equilibrium saturation state.

### 4.5. Mechanical Property Testing

The mechanical properties of pure BC and BC/PHA composite films were evaluated using a Universal Tensile testing machine (TestResources, Shakopee, MN, USA). The film specimen was tested with a pulling speed of 10 mm/min. The tensile testing data were collected for analysis of various mechanical properties (repeated three times).

### 4.6. Microstructure Analysis

The morphology and size of the 2 × 2 cm^2^ BC and BC/PHA membrane were characterised using a scanning electron microscope (SEM) (Prisma E, Thermo Scientific, Waltham, MA, USA). Before imaging, the membrane samples were sputter-coated with gold. The images were taken at an accelerating voltage of 5 kV with magnifications of 1000×.

### 4.7. Attenuated Total Reflection Fourier-Transform Infrared Spectroscopy (ATR-FTIR)

The film samples were subjected to examination using the ATR-FTIR technique. Scanning was performed at a resolution of 4 cm^−1^ with 64 scans across a wavenumber range spanning from 400 to 4000 cm^−1^. This analysis was carried out using the FT-IR spectrometer (model: Nicolet 6700) from Thermo-Scientific, Waltham, MA, USA. The recorded IR spectra captured the characteristic peaks.

### 4.8. Life Cycle Assessment (LCA)

**Figure 9:**
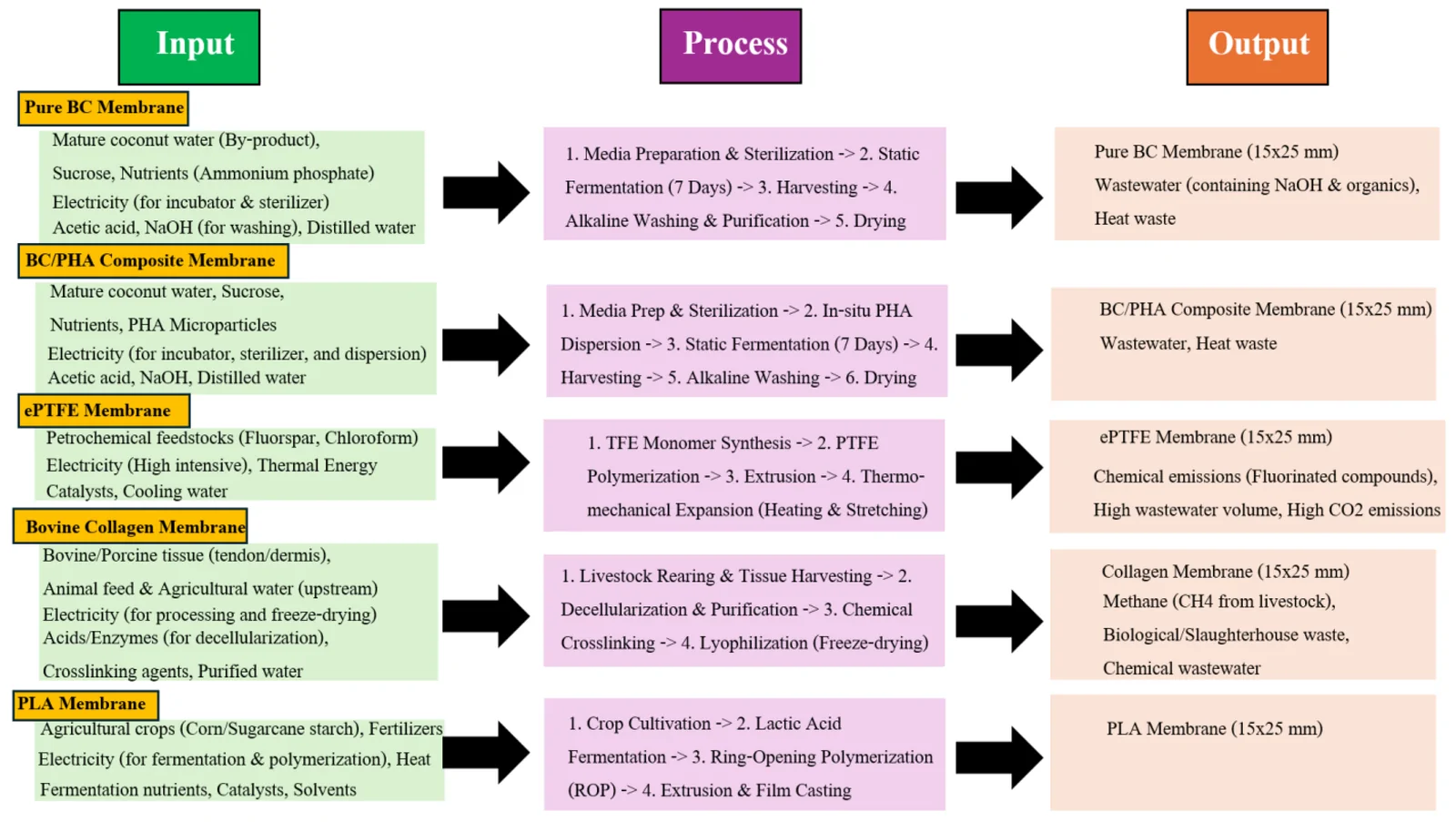
Assessment boundaries of the cradle-to-gate life cycle assessment for the Guided Tissue Regeneration (GTR) composite membrane production, encompassing bacterial cellulose synthesis and polyhydroxybutyrate production.

The life cycle assessment (LCA) was conducted in accordance with ISO 14040 and 14044 standards, adopting a cradle-to-gate assessment approach. The objective of this study was to evaluate and compare the environmental impacts associated with the synthesis and fabrication of a Guided Tissue Regeneration (GTR) membrane. The functional unit was defined as one functionally stable, suturable, and structurally intact 15 × 25 mm membrane construct possessing adequate mechanical properties for clinical application. Five distinct material scenarios were evaluated to represent both the experimental biomaterials and the prevailing commercial alternatives: (1) a pure bacterial cellulose (Pure BC) baseline synthesized via static fermentation using mature coconut water; (2) the optimized in situ composite membrane integrating polyhydroxyalkanoate (BC/PHA); (3) a commercial non-resorbable expanded polytetrafluoroethylene (ePTFE) membrane representing the synthetic petrochemical baseline; (4) a commercial resorbable bovine collagen membrane representing the natural livestock-derived baseline; and (5) a synthetic resorbable polylactic acid (PLA) membrane. The production processes and assessment boundaries for the experimental methods are illustrated in Figure 9. For the BC and BC/PHA scenarios, the inventory data encompassing the exact mass of agro-industrial raw materials, chemical reagents, and the electricity consumed during fermentation, in situ PHA dispersion, and purification were collected from primary laboratory-scale measurements. Secondary inventory data for the ePTFE, collagen, and PLA scenarios were retrieved from the Ecoinvent database version 3.9.1 and the relevant literature as listed in Table 7. The environmental impacts were analyzed using SimaPro 10.4 paired with ecoinvent 3.12 software. The assessment employed the IMPACT World+ Midpoint methodology to evaluate critical indicators, particularly global warming potential (GWP) and non-renewable energy use (NREU).

### 4.9. Statistical Analysis

A central composite design (CCD) with two independent factors, sucrose concentration and cultivation period, was employed to model their individual and interactive effects on the structural and mechanical responses of bacterial cellulose. The design comprised 13 experimental runs, including five replicated centre points (50 g/L sucrose, 7-day cultivation) to estimate experimental error and assess model reproducibility. Response surface methodology was applied to optimize the experimental data using Minitab statistical software version 16 (Minitab Inc., State College, PA, USA). Comparisons of equilibrium water content and tensile properties (ultimate tensile strength, Young’s modulus, and elongation at break) among the experimental groups were evaluated by ANOVA using SPSS Statistics version 26 (IBM Corp., Armonk, NY, USA), with statistical significance set at *p* < 0.05. All measurements were performed in triplicate, and results are expressed as mean ± standard deviation.

## Figures and Tables

**Table 1:** Treatments for optimizing BC for cellulose production.

Treatment	Factors		Variables			
	Xa	Xb	WW	DW	TW	TD
1	45	5	3.44 ± 0.08	0.23 ± 0.11	0.96 ± 0.16	0.17 ± 0.04
2	50	5	3.70 ± 0.02	0.16 ± 0.07	0.95 ± 0.09	0.11 ± 0.04
3	55	5	4.59 ± 0.26	0.18 ± 0.05	0.85 ± 0.13	0.07 ± 0.01
4	45	7	12.80 ± 0.48	0.37 ± 0.02	1.51 ± 0.33	0.09 ± 0.01
5	50	7	9.04 ± 0.23	0.24 ± 0.30	1.23 ± 0.14	0.05 ± 0.01
6	50	7	9.37 ± 0.25	0.24 ± 0.01	1.24 ± 0.15	0.07 ± 0.01
7	50	7	9.85 ± 0.07	0.23 ± 0.01	1.22 ± 0.10	0.06 ± 0.01
8	50	7	9.01 ± 0.07	0.25 ± 0.16	1.35 ± 0.14	0.06 ± 0.01
9	50	7	9.07 ± 0.17	0.26 ± 0.06	1.37 ± 0.15	0.06 ± 0.01
10	55	7	12.72 ± 0.14	0.36 ± 0.01	1.76 ± 0.21	0.09 ± 0.04
11	45	9	9.97 ± 0.30	0.76 ± 0.02	2.42 ± 0.41	0.18 ± 0.03
12	50	9	14.15 ± 0.06	0.32 ± 0.01	1.88 ± 0.19	0.07 ± 0.01
13	55	9	13.20 ± 0.19	0.54 ± 0.04	2.41 ± 0.32	0.10 ± 0.02

Note: Xa = Carbon Source (g/L); Xb = Time (days); WW = Wet Weight (g); DW = Dry Weight (g); TW = Thickness Wet (mm); TD = Thickness Dry (mm).

**Table 2:** Treatments for optimization of bacterial cellulose for mechanical performance.

Treatment	Factors		Variables		
	Xa	Xb	US	EL	YM
1	45	5	29.82 ± 2.09	36.71 ± 8.50	46.54 ± 20.14
2	50	5	14.47 ± 3.52	21.82 ± 1.92	40.57 ± 10.99
3	55	5	25.94 ± 6.50	20.03 ± 3.00	127.49 ± 72.82
4	45	7	35.23 ± 6.88	18.78 ± 5.45	249.77 ± 8.85
5	50	7	32.18 ± 4.60	15.27 ± 1.78	286.23 ± 43.66
6	50	7	29.07 ± 8.83	16.04 ± 1.18	198.58 ± 98.24
7	50	7	34.64 ± 3.89	15.53 ± 1.24	323.94 ± 65.06
8	50	7	31.28 ± 5.48	14.13 ± 2.57	275.04 ± 105.98
9	50	7	30.38 ± 11.85	15.87 ± 2.98	223.83 ± 27.21
10	55	7	44.00 ± 6.79	17.32 ± 1.69	289.79 ± 49.25
11	45	9	33.68 ± 5.72	20.64 ± 1.28	252.77 ± 33.94
12	50	9	36.05 ± 7.50	18.75 ± 5.21	242.18 ± 28.47
13	55	9	49.87 ± 2.39	17.83 ± 0.67	334.62 ± 24.04

Note: Xa = Carbon Source (g/L); Xb = Time (days); US = Ultimate Strength (MPa); EL = %Elongation; YM = Young’s Mudulus.

**Table 3:** The experimental design and the independent variable in the Box–Behnken design for the production of bacterial cellulose.

Independent Variables		Levels	
	−1	0	1
Sucrose (g/L)	45	50	55
Bioproduction period (days)	5	7	9

**Table 4:** Predictive performance of structural and mechanical model across 13 treatments.

Response Matrix	Experimental Means (Max)	Predicted Value (at Same Point)	Mean Absolute Error	Max Predicted Error	R^2^ Value
Ultimate Strength (MPa)	34.64 ± 3.89	31.11	1.55	3.53	0.9483
Young’s Modulus (MPa)	323.94 ± 65.06	256.13	22.56	67.81	0.8927
Wet Thickness (mm)	2.42 ± 0.41	1.12	0.10	0.23	0.9451
Wet Weight (g)	14.15 ± 0.06	11.70	1.01	2.45	0.8693
Dry Weight (g)	0.76 ± 0.02	0.64	0.04	0.12	0.8881
Dry Thickness (mm)	0.18 ± 0.03	0.14	0.01	0.04	0.7691

**Table 5:** Mechanical performance of commercially available alternatives in comparison to this study BC/PHA [32].

Cellulose	Maximum Tensile Stress (MPa)	Maximum Tensile Strain (%)	Elastic Modulus (MPa)
Bio-Gide	4.80	46.80	15.70
Jason	13.00	17.90	178.90
Collprotect	13.10	16.30	158.50
BC/PHA	33.76	28.20	177.51

**Table 6:** Life cycle impact assessment (LCIA) results of Guided Tissue Regeneration (GTR) composite membrane production.

Impact Category	Unit	Pure BC Membrane	BC/PHA Composite Membrane	ePTFE Membrane	Bovine Collagen Membrane	PLA Membrane
Global warming potential	kg CO_2_eq	0.0267	0.0267	0.0177	0.0864	0.00474
Non- renewable energy use	MJ deprived	0.362	0.363	0.144	0.554	0.0672

**Table 7:** Life cycle inventory data for the comparative production of five Guided Tissue Regeneration (GTR) membranes based on a functionally stable 15 × 25 mm unit.

Material/Energy	Unit/ Functional (15 × 25 mm)	Pure BC Membrane	BC/PHA Composite Membrane	ePTFE Membrane	Bovine Collagen Membrane	PLA Membrane
Inputs: Substances						
Mature coconut water	mL	37.5	37.5	-	-	-
Sucrose	g	1.875	1.875	-	-	-
Diammonium phosphate (DAP)	g	0.112	0.112	-	-	-
Glacial acetic acid	mL	0.375	0.375	-	-	-
Polyhydroxyalkanoate (PHA) microparticles	g	-	0.005	-	-	-
Precursor resin: Polytetrafluoroethylene (PTFE)	g	-	-	0.06	-	-
Raw bovine tissue	g	-	-	-	2.5	-
Precursor resin: Polylactic acid (PLA)56	g	-	-	-	-	0.06
Cleaning agent: Sodium hydroxide (NaOH)	g	0.05	0.05	-	-	-
Extraction chemicals and industrial solvents	g	-	-	0.01	-	0.01
Decellularization chemicals: Pepsin and HCl	g	-	-	-	0.5	-
Purified water for media and washing	mL	50	50	-	500	-
Cooling water for industrial machinery	mL	-	-	20	-	15
Inputs: Energy						
Electricity: Lab scale (incubator and sterilizer)	kWh	0.15	0.165	-	-	-
Electricity: Continuous industrial scale	kWh	-	-	0.01	-	0.005
Electricity: Lyophilization process	kWh	-	-	-	0.039	-
Thermal energy: Extrusion and forming process	MJ	-	-	0.05	-	0.02
Outputs: Waste Generation						
Wastewater: Total process fluid volume	mL	80.375	80.375	20	500	15
Water vapor: Loss via evaporation and drying	mL	7.5	7.5	-	-	-
Solid waste: Membrane trimmings	g	0.01	0.01	0.01	-	0.01
Solid waste: Biological fragments from processing	g	-	-	-	2	-
Emissions to water: Dissolved organics/chemicals	g	1.977	1.982	0.01	0.95	0.01

## Data Availability

Data will be made available on request.
